# Sagittal plane fore hoof unevenness is associated with fore and hindlimb asymmetrical force vectors in the sagittal and frontal planes

**DOI:** 10.1371/journal.pone.0203134

**Published:** 2018-08-29

**Authors:** Sarah Jane Hobbs, Sandra Nauwelaerts, Jonathan Sinclair, Hilary M. Clayton, Willem Back

**Affiliations:** 1 Centre for Applied Sport and Exercise Sciences, University of Central Lancashire, Preston, United Kingdom; 2 Department of Biology, University of Antwerp, Wilrijk, Belgium; 3 The Antwerp Zoo Centre for Research and Conservation, Antwerp, Belgium; 4 Sport Horse Science, Mason, Michigan, United States of America; 5 Department of Equine Sciences, Faculty of Veterinary Medicine, Utrecht University, Utrecht, The Netherlands; 6 Department of Surgery and Anaesthesia of Domestic Animals, Faculty of Veterinary Medicine, Ghent University, Ghent, Belgium; University of Illinois, UNITED STATES

## Abstract

Asymmetry in forelimb dorsal hoof wall angles, termed unevenness, is associated with forelimb gait asymmetries, but compensatory mechanisms and out of plane ground reaction forces (GRFs) due to unevenness have yet to be documented. The aim of this study was therefore to investigate the effects of fore hoof unevenness on contralateral fore and hind limb force vectors patterns, in both sagittal and frontal planes. A group of n = 34 riding horses were classified into four groups: hoof angle difference of more than 1.5 degrees (UNEVEN; n = 27), including higher left fore (HIGH-LF; n = 12), higher right fore (HIGH-RF; n = 15), and hoof angle difference of less than 1.5 degrees (EVEN; n = 7). Three dimensional ground reaction forces GRFs were collected during trotting. GRF summary vectors representing the magnitude (VecMag) and angular direction (VecAng) of the entire stance phase in the sagittal and the frontal plane were calculated. The effects of unevenness on GRF production were explored using linear regression, repeated measures ANOVA and statistical parametric mapping (SPM) with significance at (P<0.05). In all uneven groups, increasing unevenness affected sagittal VecAng values in the forelimbs, with more propulsive GRF in the high hoof. In the HIGH-RF group, medial GRFs were also found in the high RF hoof compared to lateral GRFs in the low LF hoof (RF VecAng: 0.97±1.64 (deg); LF VecAng: -0.64±1.19 (deg); P<0.05). In both HIGH groups, compensatory associations to increasing unevenness were only found in the RH, but also a significantly greater lateral VecAng was found in the LH (P<0.05) compared to the RH limb. No significant differences (P>0.05) were found between hindlimb pairs in the EVEN group. Unbalanced sagittal and increased frontal plane GRFs in uneven horses suggest that they have greater locomotory challenges, as the equine musculoskeletal system is not constructed to withstand movement and loading in the frontal plane as effectively as it is in the sagittal plane.

## Introduction

Structural and functional asymmetries between the left and right sides of the body are a part of the normal biological variation. In horses, morphological differences between the left and right limbs have been reported in bone size [1,2], muscular development [3] and hoof dimensions [4,5]. Asymmetries include unevenness, best defined by a difference in dorsal hoof wall angle of the fore hooves [6], and have been found in 5.3% of Dutch Warmblood horses [7] and can result in earlier retirement of elite sport horses [8]. The development of unevenness may be a consequence of sidedness [4,9], as has been associated with lateralized grazing posture in Warmblood horses with long limbs and short heads [9]. Asymmetrical gait patterns [9] and inter-limb differences in ground reaction force (GRF) distribution [6] are also found in these horses.

The functional consequences of unevenness are reported to be similar to sub-clinical lameness results, as asymmetrical peak vertical forces are evident between forelimbs [6]. In the lame horse, asymmetric GRFs are generally assumed to reflect a lame horse’s efforts to redistribute the weight from the lame limb to the other limbs whilst maintaining forward speed over a stride [10,11]. As such, in forelimb lameness the vertical impulse decreases in the lame forelimb and ipsilateral hindlimb, while increasing in the contralateral forelimb and diagonal hindlimb during trotting [12]. Asymmetric forelimb loading in uneven horses may induce compensatory hindlimb loading similar to that of a lame horse. Alternatively, hindlimb loading patterns may be more indicative of morphological or preferential differences that influence hindlimb function. Maintaining dynamic equilibrium through a stride must also be a factor in determining the load distribution patterns between limbs [13]. This involves balancing the forces between the limbs and may include forces outside of the sagittal plane that play a role in general locomotor stability of the complete musculoskeletal system (i.e. limbs, neck and back), as is described in hexapedal runners [14]. If hindlimb force patterns produced by uneven footed horses do vary due to preferential or morphological differences, then compensation must still allow equilibrium to be achieved in order to maintain a symmetrical and regular gait pattern.

Grouping populations of horses based on directional asymmetric biases (such as grouping by higher compared to lower peak forelimb GRFs) has recently been found to obscure differences in longitudinal GRF patterns between left and right sides [15]. Additionally, analysis of discrete variables can miss important differences in the force-time curve that occur at other time points during the stance phase. This was addressed by [16] studying the centre of pressure (COP) path under individual hooves throughout stance, described as a holistic measure of individual limb mechanics. In that study, asymmetries in dorsal hoof wall angle did not necessarily result in asymmetries in COP path, but each hoof consistently maintained its own ‘locomotor -kinematic and kinetic- fingerprint’. As such, studying the COP path was considered more suited to tracking changes over time within the individual horse.

In order to study the full extent of the force-time curve in asymmetrically oriented horses two alternative methods were recently recommended, (1) vector analysis and (2) statistical parametric mapping (SPM) [15]. The vector technique combines an easily interpretable force vector diagram with calculation of two summary variables; the vector magnitude (VecMag) represents the force magnitude over the entire stance phase and is influenced primarily by the vertical GRF, while the vector angle (VecAng) represents the direction of the GRF and is influenced by the horizontal force components. To complement the vector technique which provides visual comparisons and summary variables, SPM can be used to objectively identify significantly different regions between multi-dimensional, time-continuous GRF profiles [17,18].

As the presentation of unevenness may be important to orthopaedic health and the compensatory mechanisms used by uneven footed horses are currently unknown, the aim of this study was to investigate the effects of limb specific (left vs right) and directionally-biased (high vs low) fore hoof unevenness on contralateral fore and hind limb force vectors patterns, in both sagittal and frontal planes. This study applies vector analysis and SPM to explore the extent of the GRF patterns in uneven footed horses with known functional asymmetries in the forelimbs.

The objectives were 1) to seek associations between direct (known) and compensatory (unknown) functional GRF asymmetries and asymmetries in dorsal hoof wall angles in uneven horses grouped using directional-bias (UNEVEN; high vs low) and additionally by limb specific morphology (a higher dorsal hoof wall angle in the left (HIGH-LF) or right (HIGH-RF) forelimbs), and 2) to compare the GRF vector patterns produced by contralateral fore and hind limbs in uneven footed horses grouped by directional bias (UNEVEN), uneven footed horses grouped laterally (HIGH-LF and HIGH-RF) and even (EVEN) footed horses, using the techniques of force vector analysis and SPM.

For this study, we developed the following hypotheses: Sagittal plane force vector patterns in the forelimbs would be influenced by the degree of asymmetry of the fore hooves, based on the findings of a difference in vertical and longitudinal forelimb GRFs in uneven footed horses [6]. Grouping the horses by directional bias would reduce the significance of any horizontal GRF asymmetries, based on the findings of [15]. Patterns of HIGH-LF and HIGH-RF diagonal pairs patterns would be mirror images, as morphological asymmetry results in similar locomotor asymmetry in the forelimbs independent of side [6,9]. Finally, that compensatory GRF patterns in the hindlimbs would subtly follow those described by [12] in lame horses, as forelimb GRF patterns in uneven footed horses are similar to sub-clinical lameness patterns [6].

## Materials and methodology

This study was performed in accordance with Dutch law. A formal waiver of ethics approval was granted by the Animal Welfare Body Utrecht in 2011. The waiver was granted as the study was non-invasive and all horses were either client-owned, in which case the owners consented to the study, or they were school horses belonging to the university. UCLan Animal Welfare and Ethics Review Board (AWERB) did not prospectively review the project in 2011, as there was no formal requirement for UCLan staff involved in overseas projects to apply for formal approval at that time. In 2014, approval was granted by UCLan AWERB for a generic non-invasive procedure (Ref: REPROC/14/01/SH) and can confirm that techniques used in the Dutch 2011 project fall within that procedure.

This study followed the protocol for multi-dimensional time GRF vector analysis dx.doi.org/10.17504/protocols.io.r3dd8i6 [PROTOCOL DOI].

The subjects were n = 27 uneven footed (dorsal hoof wall angle difference > 1.5 degrees, as classified by [6]) and n = 7 even footed riding horses of different breeds (mean±SD, bodyweight: 557±77 kg; age: 12 ± 5 years). The horses were evaluated by an experienced, board certified clinician on straight lines at walk and trot, which constitutes part of a lameness examination. The horses were graded on a scale of 0–5 according to the American Association of Equine Practitioners lameness scale [19] for each gait separately. As such, some of the horses were 0–0: sound at walk and trot, while some in the uneven groups were 1–0: 1/5 lame at walk (asymmetrical head nod) and sound at trot (symmetrical head nod) [6], see Table 1. In addition, the absolute difference in peak vertical GRF (%) between left and right limbs for the forelimbs and hindlimbs was determined at the trot retrospectively (Table 1).

**Table 1:** Assignment of horses according to subjective lameness evaluation and % difference in absolute peak vertical GRF between left and right limbs during trotting.

		Lameness grade (walk-trot)		% difference in peak GRF during trotting	
		0–0	1–0	Forelimbs	Hindlimbs
HIGH-LF	n	7	5	4.1 (2.5)	4.2 (2.3)
Hoof angle difference (deg)		4.8 (2.0)	4.8 (3.0)		
HIGH-RF	n	11	4	4.4 (4.0)	3.2 (4.0)
Hoof angle difference (deg)		4.8 (3.8)	4.9 (2.3)		
EVEN	n	7	0	2.7 (1.3)	6.4 (7.9)
Hoof angle difference (deg)		0.73 (0.51)			

Number of horses and mean (SD) hoof angles of hooves classified according to the fore hoof (LF,RF) with the higher dorsal hoof wall angle (HIGH-LF, HIGH-RF, EVEN) and according to lameness grade evaluated on a scale of 0 to 5 at walk and trot (American Association of Equine Practitioners, 2017).

### Data collection

Six retroreflective markers were attached to each hoof at the level of the coronet and distal hoof wall mid-dorsally, and laterally and medially at the widest part of the hoof. These markers were used to locate the position of the hoof on the force plate and to determine the position of the COP relative to hoof position. The hoof markers were tracked by 8 Oqus 3+ cameras operating at 250 Hz and processed using Qualisys Track Manager (QTM version 2.1). Ground reaction forces (GRF) were measured using a force platform (Kistler Z4852C, 60 × 90 cm), which captured data at 1000 Hz.

A trial captured with the horse standing square was used to determine the dorsal hoof wall angles of each fore hoof by measuring the angle between the line joining the proximal and distal markers on the dorsal hoof wall to the horizontal. The difference between LF and RF dorsal wall angles was determined and the horses were firstly categorized into EVEN and UNEVEN groups. The UNEVEN horses were further categorized into two asymmetry groups: left fore higher: HIGH-LF; right fore higher: HIGH-RF. High and low hoof angles were compared between the HIGH-LF and HIGH-RF groups using an independent samples t-test prior to further analysis to establish whether unevenness was similar between groups.

The horses were trotted in hand along a rubberized runway with an embedded pressure and force plate until a minimum of 3 clean hits had been recorded for each of the four limbs at a consistent velocity.

### Data processing

Kinematic data was tracked in QTM and then exported into Visual 3D (version 5.02). Thresholds of above and below 50 N of vertical GRF were used to define the start and end of the stance phase respectively. The velocity of each trial was calculated as stride length divided by stride duration using the kinematic markers on the hooves post processing. For sagittal plane data, the cranio-caudal (C-C) direction was positive in the direction of movement. For frontal plane data, the medio-lateral (M-L) direction was positive medially, i.e. when viewing from the rear, to the right for the left limbs and to the left for the right limbs. GRF trials of LF and RF were matched based on a velocity difference of less than 0.1 m·s^-1^, which yielded a total of 1 (n = 6), 2 (n = 16) or 3 (n = 12) trials of matched data per horse.

The GRF data were normalized to horse mass, down-sampled to 250 Hz and plotted as vector diagrams in all three planes of motion [15]. The summary variables VecMag and VecAng were determined in the sagittal and frontal planes. VecMag was calculated by vector summation of the individual vectors divided by the number of samples contributing to the value, and VecAng was determined trigonometrically from the components of the vector magnitude and expressed relative to the vertical with positive values being directed cranially in the sagittal plane and medially in the frontal plane. Between-limb differences in hoof angle, VecMag and VecAng were calculated separately for the sagittal and frontal plane data by subtracting the values for the hoof with the lower dorsal wall angle from those of the hoof with the higher angle. Mean values of the vector variables were calculated for each horse with more than 1 matched trial. For horses with a single matched trial, the values for that trial were used in the analysis. Differences in hoof angles and vector variables, together with absolute values of the vector variables for each limb, were tabulated in Excel (version 2007/2016) and then imported into SPSS (version 24).

#### Relationship between vector variables and dorsal hoof wall angle

Forward stepwise multiple linear regression was performed for the UNEVEN, HIGH-LF and HIGH-RF groups separately to 1) determine the strongest association between dorsal hoof wall angle asymmetry and vector variables (direct relationship to unevenness); and 2) from these results, determine the strength of the relationship to other vector variables that may indicate compensatory effects (relationship with the key functional consequences of unevenness). Tolerance and variance inflation factor (VIF) were extracted to assess co-linearity between predictor variables in the model.

#### Differences in GRF patterns between contralateral limb pairs

For the vector summary variables, contralateral limb pairs were compared using repeated measures ANOVA separately for the UNEVEN, HIGH-LF, HIGH-RF and EVEN groups, with significance indicated at p<0.05.

Where significant differences (p<0.05) were found in ANOVA results, SPM analysis was conducted post-hoc to explore the temporal patterns further. For SPM analysis, the stance phase GRF data in all three dimensions (vertical, longitudinal and mediolateral axes) from the horses in each group with significant findings were normalized to total mass (N/kg) and the duration of the stance phase was similarly normalized to 101 data points. These variables were assembled into vector fields of # horses, 101 data points per stance phase and three dimensions per data point for each limb (UNEVEN, 27*101*3; HIGH-LF, 12*101*3; HIGH-RF, 15*101*3). Differences between contralateral fore and hind limb pairs, were examined for each group separately via a statistical parametric mapping approach using MATLAB 2017a (MATLAB, MathWorks, Natick, USA), with the source code available at [20]. Statistical parametric mapping was implemented with planned comparisons in a hierarchical manner. Specifically, Hotelling’s T2 tests were used to compare the vertical, longitudinal and mediolateral continuous data together, followed by individual paired t-tests on each GRF dimension.

## Results

In the uneven horses, the differences between dorsal hoof wall angles in the forelimbs ranged from ≥1.5 to 12.3 degrees. In the even horses the difference ranged from 0.2 to <1.5 degrees. The profiles of each lateral asymmetry group (HIGH-LF, HIGH-RF), and symmetrical group (EVEN) together with details of the lameness scores are shown in Table 1. There was no significant difference in the unevenness of the HIGH groups (Low dorsal wall angle: HIGH-LF = 48±3, HIGH-RF = 50±5 deg, P = 0.135; High dorsal wall angle: HIGH-LF = 53±4, HIGH-RF = 55±5 deg, P = 0.150).

### Relationship between vector variables and dorsal hoof wall angle

**Fig 1:**
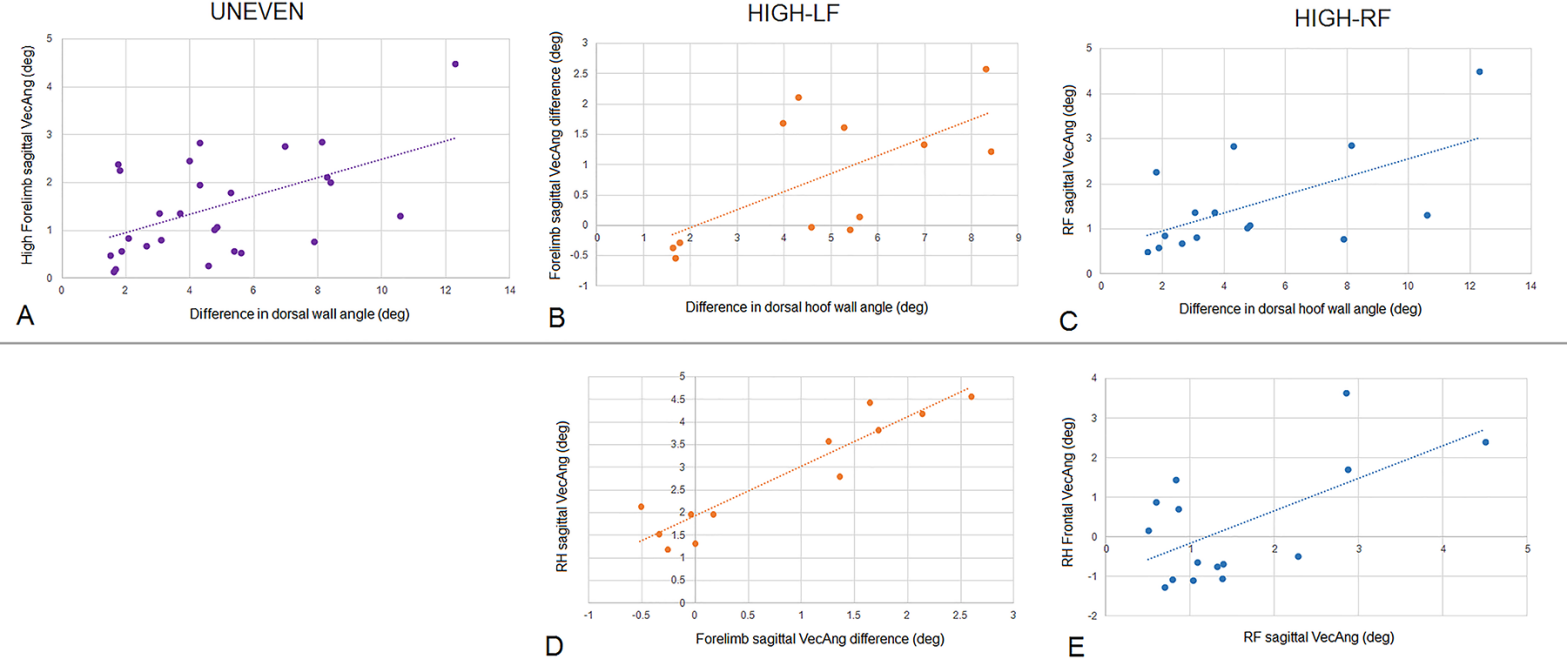
Plots of regression analysis results for UNEVEN, HIGH-LF and HIGH-RF groups. A-C Results of initial analysis of dorsal hoof wall angle difference against summary vector variables. D-E Results of secondary analysis of initial outcome variables against remaining summary vector variables.

Linear regression analysis was used to seek associations between the difference in forelimb dorsal hoof wall angles with the vector summary variables in the sagittal and frontal planes (Table 2, Fig 1). In the UNEVEN group, hoof angle difference was positively related to the high hoof sagittal VecAng (R = 0.531; P = 0.004). In the HIGH-LF group, hoof angle difference was positively related to forelimb sagittal VecAng difference (R = 0.645; P = 0.024). In the HIGH-RF group, hoof angle difference was positively related to RF sagittal VecAng (R = 0.601; P = 0.018). No other variables were included in these models.

**Table 2:** Results of linear regression analysis of difference in dorsal hoof wall angles tested against vector summary variables in the sagittal and frontal planes using forward stepwise linear regression.

	R	R^2^	B	S.E.B	β	p
UNEVEN						
Model 1	0.531	0.282				
Constant			2.626	0.849		
High Forelimb Sagittal VecAng			1.471	0.469	0.531	0.004
HIGH-LF						
Model 1	0.645	0.416				
Constant			3.685	0.689		
Forelimb Sagittal VecAng Difference			1.400	0.525	0.645	0.024
HIGH-RF						
Model 1	0.601	0.361				
Constant			2.068	1.245		
Right Forelimb Sagittal VecAng			1.805	0.666	0.601	0.018

UNEVEN (n = 27, categorized as high and low hoof angles), HIGH-LF (n = 12, higher left fore hoof) and HIGH-RF (n = 15, higher right fore hoof). Multiple correlation coefficient (R), coefficient of determination (R^2^), unstandardized regression coefficient, (B), standard error of B (S.E.B.), standardized regression coefficient (β), probability (p).

The outcome variables from the previous linear regression results were then compared to all other vector variables (Table 3, Fig 1). In the UNEVEN group, all of the variables were included in the model, consequently a clear relationship between high hoof sagittal VecAng and the remaining vector variables was not found. For the HIGH-LF group, the difference in forelimb sagittal VecAng between limbs was positively related to RH sagittal VecAng in the model (R = 0.935, P<0.001). For the HIGH-RF group, RF sagittal VecAng was positively related to RH frontal VecAng (R = 0.620, P = 0.014). No other variables were included in these models.

**Table 3:** Results of linear regression analysis from outcome variables in initial regression analysis (HIGH-LF = Forelimb Sagittal VecAng Difference; HIGH-RF = RF Sagittal VecAng) tested against remaining vector summary variables.

	R	R^2^	B	S.E. B	β	Sig
HIGH-LF						
Model 1	0.935	0.875				
Constant			-1.461	0.294		
Right Hindlimb Sagittal VecAng			0.804	0.096	0.935	< .001
HIGH-RF						
Model 1	0.620	0.384				
Constant			1.397	0.239		
Right Hindlimb Frontal VecAng			0.466	0.164	0.620	.014

The UNEVEN group is not included, as all variables were included in the model. Multiple correlation coefficient (R), coefficient of determination (R^2^), unstandardized regression coefficient, (B), standard error of B (S.E.B.), standardized regression coefficient (β), probability (p).

### Differences in GRF patterns between contralateral limb pairs

**Fig 2:**
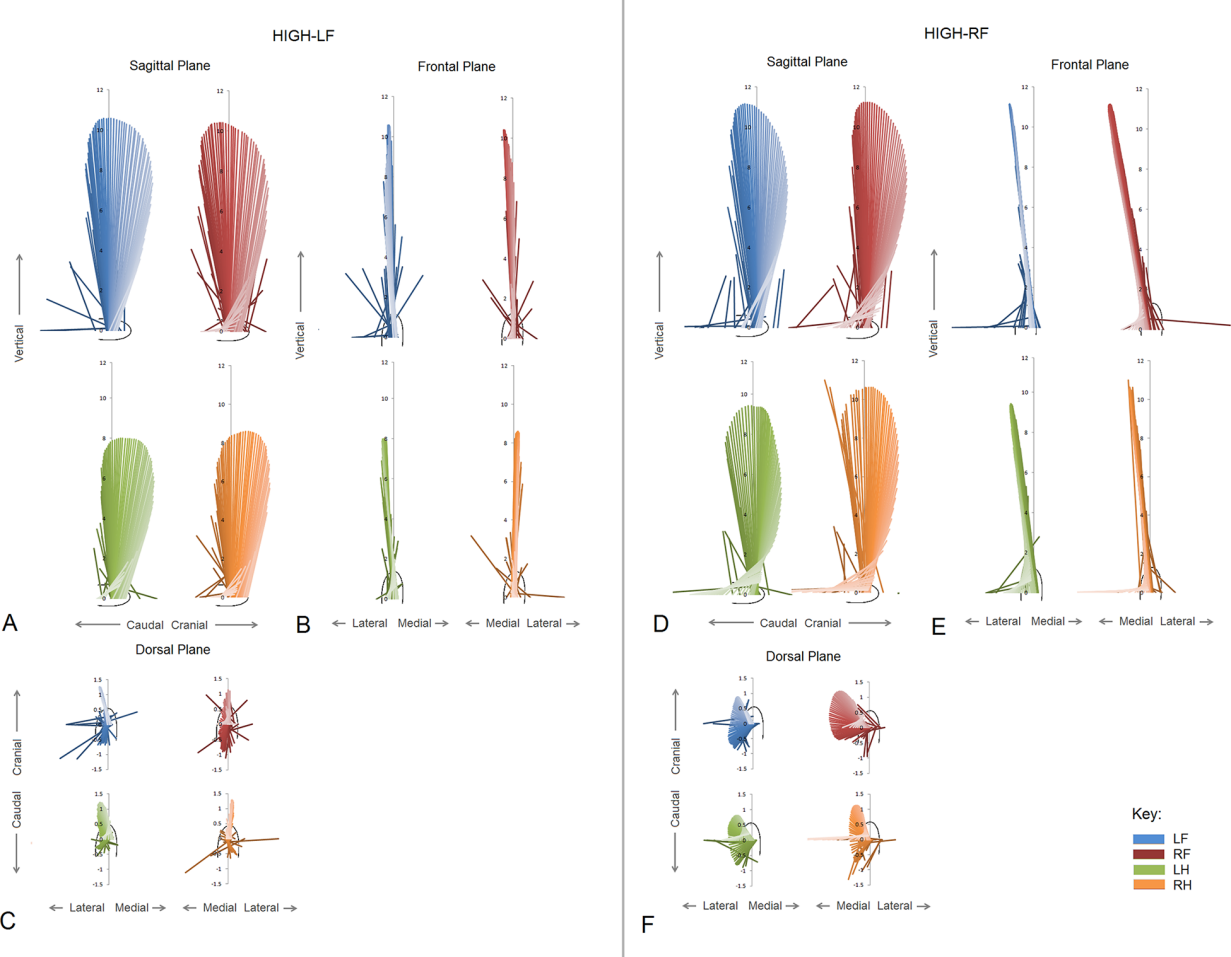
Vector diagrams of sagittal, frontal and dorsal views for one asymmetric horse in each HIGH group. HIGH-LF group (A,B,C), HIGH-RF group (D,E,F).

Force vector diagrams for the three planes of motion are illustrated for each limb of a typical uneven horse within each HIGH group in Fig 2. Summary vector variables separated by symmetry-asymmetry group are shown in Table 4. Paired comparisons between contralateral limb pairs for the UNEVEN group were significantly different for forelimb sagittal VecAng (P = 0.034) and forelimb sagittal VecMag was close to significance (P = 0.058). For the HIGH-LF group significant differences were identified for forelimb sagittal VecAng (P = 0.027) and hindlimb frontal VecAng (P = 0.044). For the HIGH-RF group, significant differences for frontal VecAng were identified in both the forelimbs (P = 0.034) and hindlimbs (P = 0.005). No significant differences (P>0.05) were found for the EVEN group.

**Table 4:** Paired comparisons of summary vector variables according to left-right and high-low differences in fore hoof angles of uneven and even footed horses.

		HIGH-LF	HIGH-RF	EVEN		UNEVEN
	n	12	15	7		27
Forelimbs						
Sagittal VecMag	L	6.21 (0.46)	6.60 (0.68)	6.31 (0.64)	Lo	6.44 (0.63)
	R	6.25 (0.53)	6.33 (0.53)	6.45 (0.53)	Hi	6.28 (0.49)
Frontal VecMag	L	6.21 (0.46)	6.60 (0.68)	6.31 (0.64)	Lo	6.44 (0.63)
	R	6.25 (0.53)	6.33 (0.52)	6.45 (0.53)	Hi	6.28 (0.49)
Sagittal VecAng	L	1.44 (1.00)*	1.27 (1.17)	1.38 (1.46)	Lo	0.99 (1.05)*
	R	0.64 (0.80)*	1.52 (1.12)	0.97 (0.53)	Hi	1.49 (1.05)*
Frontal VecAng	L	-0.60 (1.43)	-0.64 (1.19)*	-0.65 (1.71)	Lo	-0.05 (1.32)
	R	0.70 (1.10)	0.97 (1.64)*	0.42 (1.11)	Hi	0.27 (1.71)
Hindlimbs						
Sagittal VecMag	L	5.35 (0.57)	5.56 (0.54)	5.33 (0.73)	Lo	5.41 (0.54)
	R	5.44 (0.61)	5.46 (0.52)	5.60 (0.40)	Hi	5.51 (0.56)
Frontal VecMag	L	5.35 (0.57)	5.55 (0.53)	5.32 (0.73)	Lo	5.41 (0.53)
	R	5.44 (0.61)	5.45 (0.52)	5.59 (0.40)	Hi	5.50 (0.56)
Sagittal VecAng	L	2.84 (1.29)	3.55 (1.74)	3.62 (1.35)	Lo	3.04 (1.42)
	R	2.81 (1.26)	3.21 (1.53)	3.36 (1.61)	Hi	3.22 (1.56)
Frontal VecAng	L	-1.59 (0.84)*	-1.63 (1.30)*	-1.23 (1.77)	Lo	-0.56 (1.54)
	R	-0.46 (1.51)*	0.27 (1.49)*	-0.07 (1.12)	Hi	-1.11 (1.49)

Summary vector magnitudes (VecMag, N/kg) and vector angles (VecAng, degrees) in the sagittal and frontal planes separated according to asymmetry group (left fore higher: HIGH-LF; right fore higher: HIGH-RF; dorsal wall angle difference <1.5 degrees: EVEN; and dorsal wall angle difference >1.5 degrees: UNEVEN).

* indicate pairs of values that differ significantly in each column (P<0.05).

L: left; R: right; Lo: low dorsal wall angle; Hi: high dorsal wall angle.

**Fig 3:**
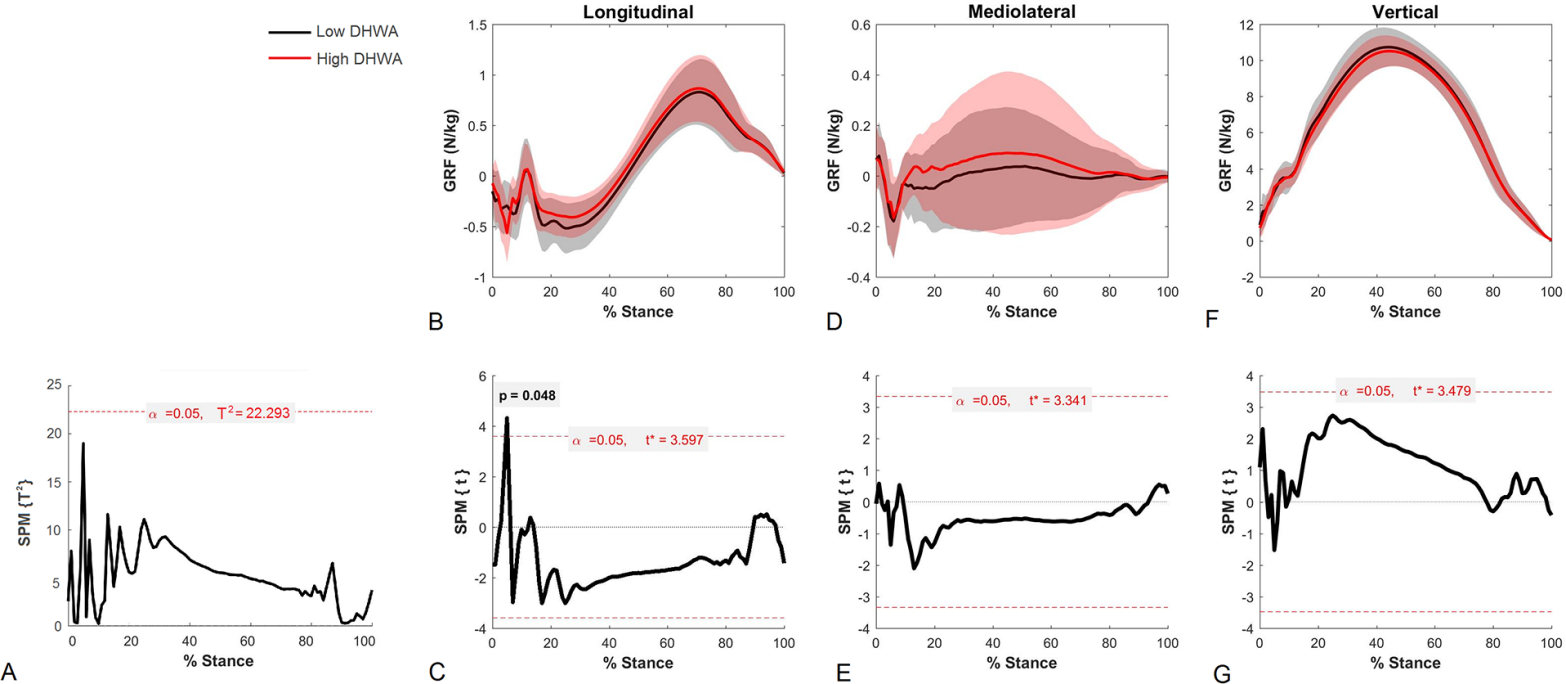
Vector analysis of the high and low forelimb GRFs in the UNEVEN group. 3D vector field results of SPM analysis (A), and 1D SPM results for individual GRF component fields (C = longitudinal, E = medio-lateral, G = vertical) and the corresponding GRF plots (N/kg) (B = longitudinal, D = medio-lateral, F = vertical) for high (red) and low (black) dorsal hoof wall angles (DHWA) for the UNEVEN group.

Post hoc results of the vector SPM analysis for the forelimbs in the UNEVEN group are shown in Fig 3. Vector field SPM analysis (vertical, longitudinal and mediolateral continuous data combined) are shown in Fig 3A. The horizontal dashed line indicates the critical threshold above which left and right T^2^ values are significantly different. T^2^ is closer to the significance threshold in the first half of the stance phase, but does not reach significance. Component GRF traces for left and right limbs together with the corresponding SPM analysis for each component separated by group are shown in Fig 3B–3G. One data point in the longitudinal direction exceeded the critical threshold during the impact phase (P = 0.048).

**Fig 4:**
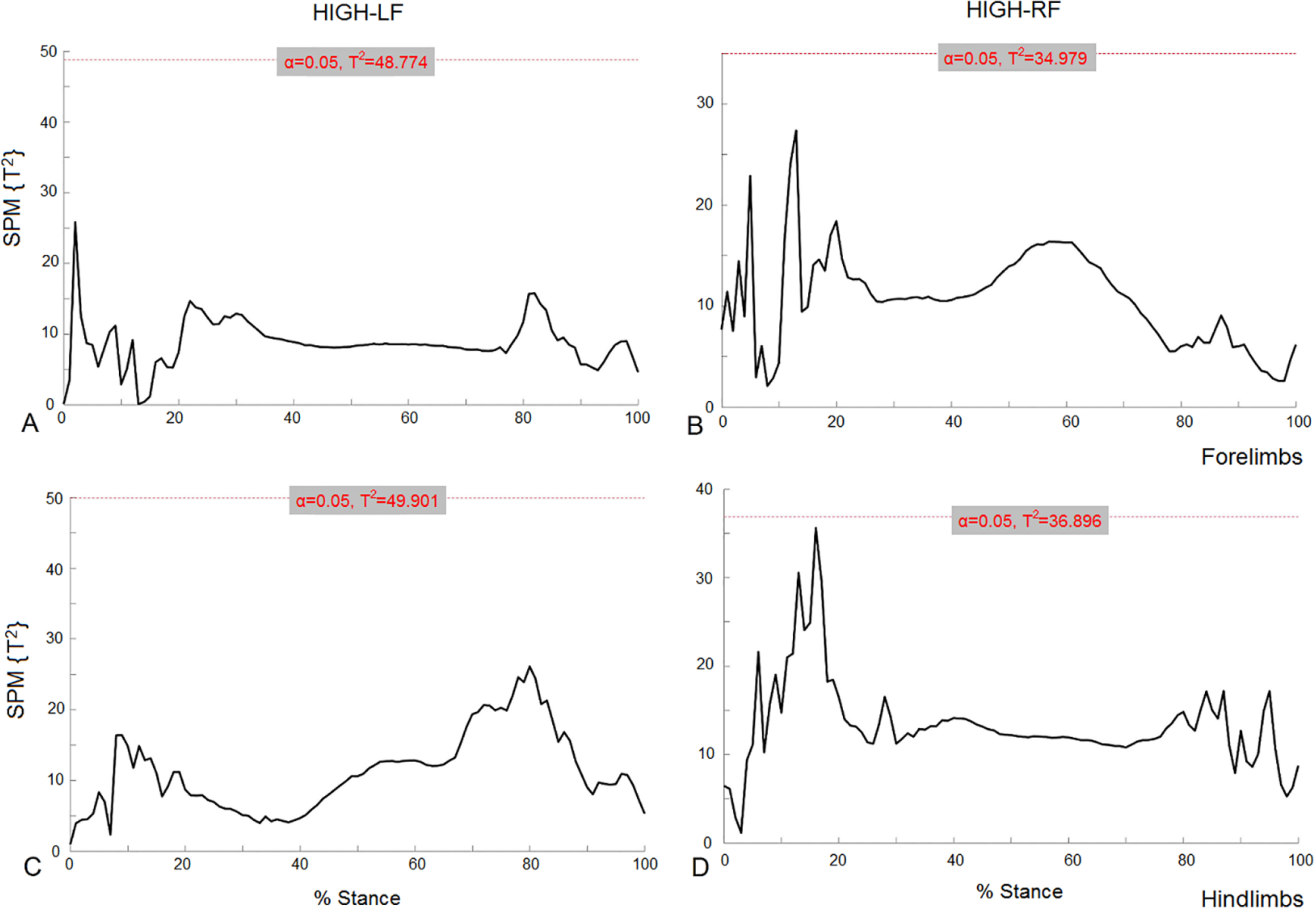
3D vector field results of SPM analysis for fore and hindlimbs for the HIGH groups. HIGH-LF (A = forelimbs, C = hindlimbs) and HIGH-RF (B = forelimbs, D = hindlimbs).

For the HIGH groups, vector field SPM analysis (vertical, longitudinal and mediolateral continuous data combined) are shown in Fig 4. In the HIGH_RF group T^2^ is closer to the significance threshold for both fore and hindlimbs in the first half of the stance phase, but as no threshold crossings were found there were no significant differences (P>0.05) in the fore or hind limbs for either the HIGH-LF or HIGH-RF groups.

**Fig 5:**
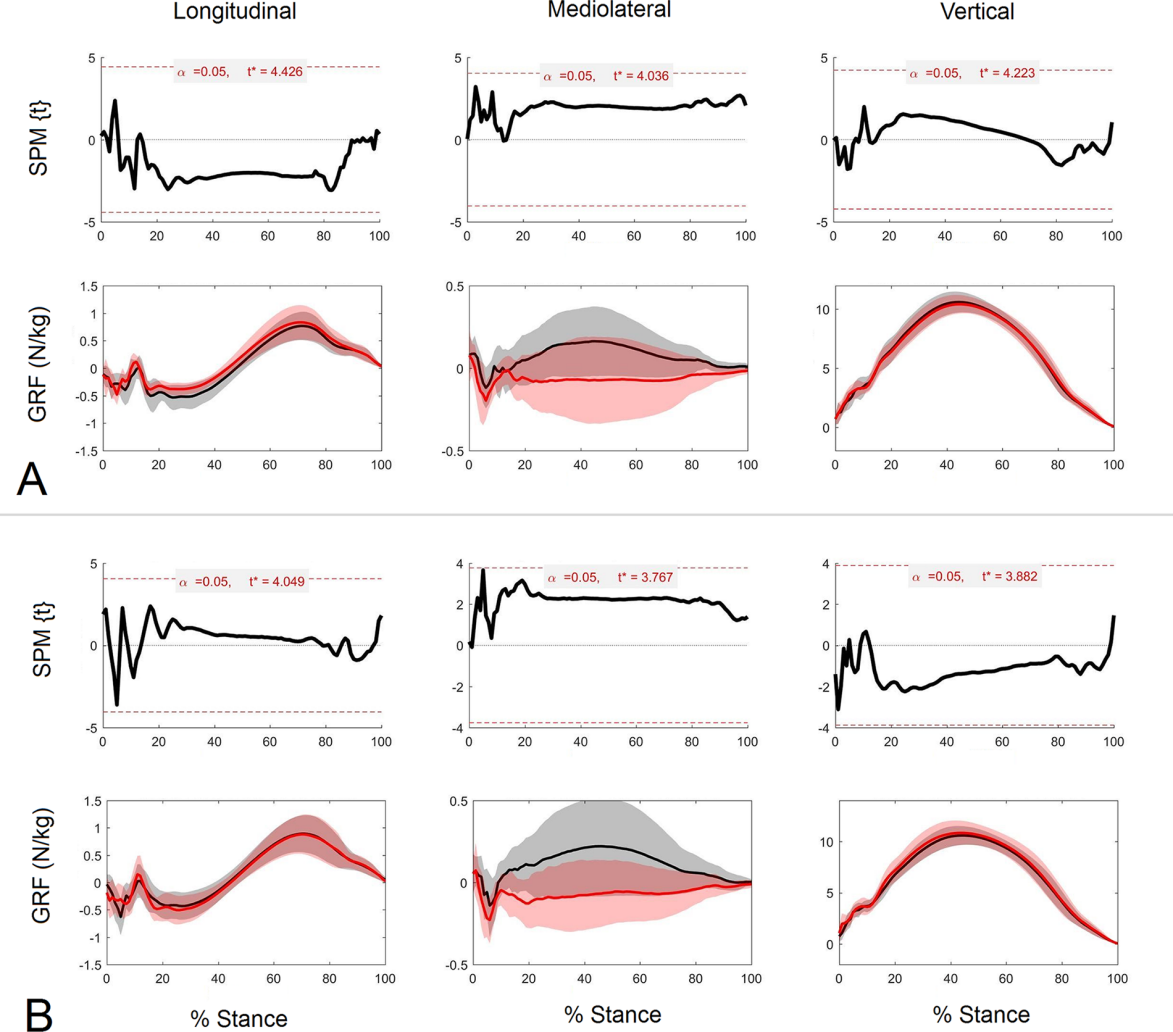
1D vector analysis of the left and right forelimb GRFs for the HIGH groups. 1D SPM results for individual GRF component fields and the corresponding GRF plots (N/kg) for left (red) and right (black) forelimbs for HIGH-LF (A) and HIGH-RF (B).

**Fig 6:**
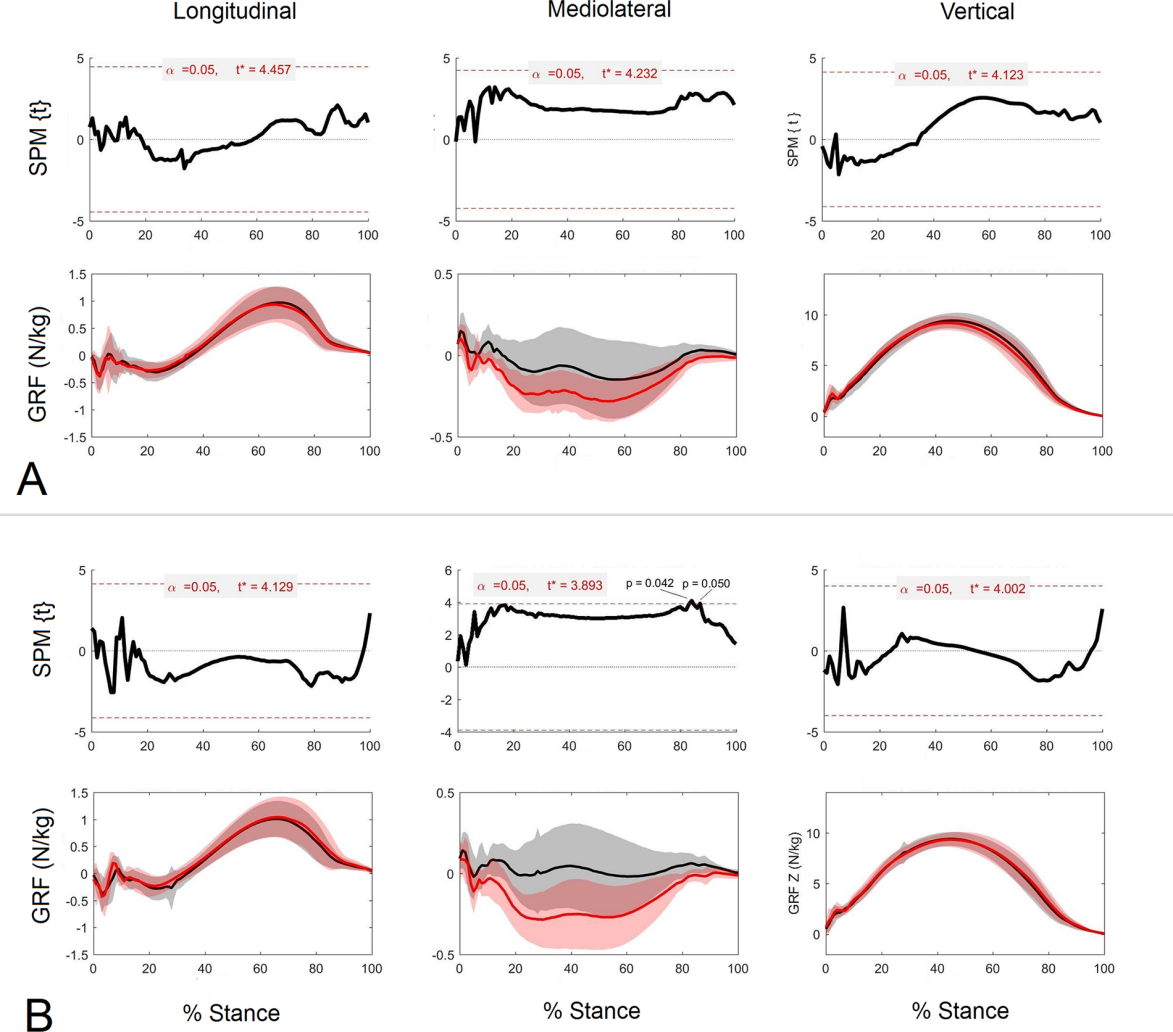
1D vector analysis of the left and right hindlimb GRFs for the HIGH groups. 1D SPM results for individual GRF component fields and the corresponding GRF plots (N/kg) for left (red) and right (black) hindlimbs for HIGH-LF (A) and HIGH-RF (B).

Component GRF traces for left and right limbs together with the corresponding SPM analysis for each component separated by group are shown in Fig 5 for the forelimbs and Fig 6 for the hindlimbs. Comparing the traces between groups in the forelimbs it is evident that the t value is closer to the threshold in the longitudinal direction for HIGH-LF, although none of the values reached significance (P>0.05). For the hindlimbs, in the HIGH-RF group SPM analysis identified a significant difference between mediolateral GRF vectors for the LH and RH (P<0.05). Two clusters of data points exceeded the critical threshold during breakover (P = 0.05; P = 0.046) and the t value was close to the threshold for the majority of the stance phase.

## Discussion

Comparison of sagittal plane force vectors displayed at intervals throughout stance has been used for several years in people under the names of Pedotti diagrams or butterfly diagrams [21–24]. This technique has recently been applied in horses using sagittal plane force vectors to calculate summary vector variables (VecMag, VecAng) to facilitate numerical analysis and by applying SPM to provide continuous statistical analysis of interlimb differences in sagittal plane GRFs throughout the stance phase [15]. The present study advances the application of quadrupedal force vector analysis by evaluating 3D force vectors in all four limbs of horses with hoof asymmetries. A previous study [6] has shown differences in forelimb peak vertical and longitudinal GRF values in horses with asymmetrical fore hooves. In this study we have used the same population of horses, grouped by dorsal hoof wall angle differences, and have applied vector analysis and SPM to explore multi-dimensional compensatory effects this time in all four limbs, to provide a method of studying direct and compensatory effects due to uneven fore hooves.

We hypothesized that sagittal plane force vector patterns in the forelimbs would be influenced by the degree of asymmetry of the fore hooves. Indeed, for the UNEVEN and the HIGH-LF group, there was a significant difference in sagittal VecAng between forelimbs and this difference had a strong relationship to the difference in dorsal hoof wall angle (Tables 2 and 4, Fig 1). Additionally, significant differences between longitudinal GRFs were found for the UNEVEN group during impact. For the HIGH-RF group, sagittal VecAng in the right forelimb was increasingly more cranially directed as the hoof wall angle asymmetry increased between forelimbs (Fig 1), but mean differences between limbs were found in the frontal and not the sagittal plane (Table 4). This hypothesis could largely be accepted and concurs with [6] who found reduced braking and an earlier transition from braking to propulsion in the higher foot, although the difference between limbs was more strongly evidenced in the UNEVEN and HIGH-LF groups. Linked to this is the second hypothesis, that grouping the horses by directional bias would reduce the significance of any horizontal GRF asymmetries. Clearly, this was not the case for the forelimbs, as sagittal VecAng and longitudinal GRFs were significantly different between high and low dorsal wall angles in the UNEVEN group. The strength of this finding supports previous literature indicating that dorsal hoof wall angles influence sagittal plane mechanics. In the hindlimbs, however, grouping in low and high diagonal pairs negates the influence of left-right differences and as such, no compensatory effects were detected in the hindlimbs in the UNEVEN group (Table 4).

The third hypothesis, that HIGH-LF diagonal pair patterns would mirror HIGH-RF diagonal pair patterns was rejected. This was evidenced by VecAng results throughout the analysis, which were not mirrored, but perhaps most strikingly by the significantly greater lateral VecAng in the left hindlimb in both groups. This may be an important finding in terms of considering orthopaedic health, as the horses’ locomotor system is not ideally designed to withstand concomitant out of sagittal plane movements. For the last hypothesis, although none of the VecMag results were significantly different between contralateral limbs, the magnitudes in all four limbs of all four groups subtly followed the peak vertical GRF patterns described for subclinical lameness [12]. As a consequence this hypothesis was neither accepted nor rejected. Although the VecMag patterns were not significantly different, they are expected to be a necessary requirement in maintaining steady state gait for a horse with an asymmetrical hoof conformation and possibly reflect preferential central locomotor steering.

The differences in VecAng patterns for each limb between HIGH groups suggest that methods of managing COM moments to achieve stability over a stride are related to the degree of unevenness and also to a limb bias. VecAng in the sagittal plane is principally indicative of longitudinal GRF production [15] and managing hindlimb propulsive GRFs is an important motor control strategy for horses performing extremely collected gaits (e.g. passage; [25]). For the HIGH-LF group, as dorsal wall angle disparity increased, an increase in propulsive VecAng was found in the high LF hoof and the diagonal RH hoof. Sagittal VecAng was also larger in the LF in the group as a whole. This indicates that as the horses in this group became more uneven, they accelerated more during the left fore-right hind diagonal step. This may be a compensatory mechanism used to maintain speed. An increase in propulsive effort was reported in horses trotting on soft beach sand to maintain speed where the overall vertical GRF was reduced [26]. However, the compensation patterns in VecMag between limbs are similar to the vertical GRF redistribution as reported in lame horses [12], so a subtle redistribution vertical GRF may also be used to maintain a steady state trot. Other locomotor deficits that would require an acceleratory step could include a limited ability to store and release elastic energy in the HIGH limb, as [6] reported an increase in stiffness of the upright HIGH limb. Out of balance, forces during a step will also influence COM moments [13], which may cause unwanted rotations in either the sagittal or frontal plane.

For the HIGH-RF group, an increase in dorsal hoof wall disparity led to an increase in propulsive VecAng in the HIGH RF hoof, together with an increase in medial VecAng the ipsilateral RH. For the RF-LH, diagonal pair there was also a mean medial VecAng in RF and a mean lateral VecAng in LH. Similarly, but at the walk, a slightly, but not significantly, more medially positioned GRF vector was found in right forelimbs compared to left forelimbs in horses walking in a straight line [27]. This was, in part, to medio-lateral hoof balance, although it was recognized that adjustments in limb placement would alter the orientation of the medio-lateral GRF vector. In the HIGH-RF group, it is also quite striking that the medio-lateral forces were mainly directed to the left side of the horse, which could increase the difficulty of turning in a clockwise direction. As such, HIGH-RF horses may derail more readily when moving on clockwise circle [28].

Based on the findings of [12] and the patterns for VecMag, the HIGH limb in both groups could be considered as the ‘affected’ limb, and postural changes in uneven footed horses, in theory, may follow the same trends as induced lameness. In a previous study [29], induction of a mild LF lameness was associated with increased thoracic range of motion (ROM) in flexion-extension, reduced thoracic and sacral ROM in lateral bending and the cranial thoracic vertebral column was bent (concave) towards the lame limb at midstance. The reduced ROM in lateral bending was expected to be due to ‘spinal stiffening’, that is increased contraction of the paraspinal muscles, and it was proposed as one of the mechanisms used to unload the lame limb [30]. In sound trotting horses *m*. *longissimus dorsi* activity occurs from mid swing of the ipsilateral hindlimb until early stance and in the propulsive phase of stance [31,32]. In order to facilitate lateral bending towards the lame limb a larger, earlier contraction on the contralateral, lame side, would be expected (i.e. for left fore lameness, earlier and larger contraction on the left side during swing of the left hindlimb). Both sides would then be active during the lame propulsive phase, which would reduce the ROM in lateral bending. If spinal stiffening occurs in uneven horses on the HIGH diagonal, then the out of balance longitudinal and medio-lateral forces together with a stiffer spine may cause a yaw rotation of the body. This is perhaps illustrated better in the HIGH-RF group, as a larger lateral GRF is produced by the left hind in late stance, which with increased spinal stiffness could push the hindquarters to the left. A correction (possibly altered limb placement) would then be required by the opposite diagonal to continue moving straight. Further work is needed to explore functional adaptations in these horses, both from a kinematic and electromyographical perspective.

The difference in forelimb vector patterns between the HIGH groups indicates that motor control is subtly influenced by which side is the HIGH side, but left hindlimb medio-lateral GRF patterns are side-independent. Lateralized grazing behavior, commonly found in uneven footed horses, has been linked to sidedness [4,9], but a relationship between jumping tasks and unevenness has not yet been found [4]. This may be because the LH dictates aspects of locomotor function, which confound the functional consequences of unevenness during specific tasks. In the largest study of handedness in horses to date, 90% of Thoroughbreds, Arabians, and American Quarter horses preferred a right lead stride pattern [33]. A population bias has not been found in relation to fore hoof unevenness to date in Warmbloods [9], but in Thoroughbred horses with unilateral club foot one study reported a prevalence of 75% in the right foot [34]. It is possible that the RF-LH diagonal is more commonly dominant in the horse population, although this is currently a speculative suggestion. Certainly, the HIGH and EVEN groups in our study all produced a laterally directed VecAng in the left hindlimb, although unevenness exacerbated the production of laterally directed GRFs. In humans that are right pelvic limb dominant, higher laterally and lower medially, directed forces and impulses have been found in the right limb during walking [35]. Larger net joint moments at the right hip, tarsal, and metatarsophalangeal joints have been found in dogs with a directional hindlimb bias [36], which was indicated as a measure of limb dominance. Such increases should be reflected in our hindlimb VecMag results if LH was a dominant hindlimb, which were not evident. A RF-LH dominance also contradicts the work of [37], who found no correlation between lateralized grazing behavior and hindlimb flexing.

As motor lateralization in horses is reported to be more prevalent with age [28,37], the GRF patterns we observed in the LH may be a training effect. Handler position may also have been a factor, although care was taken not to influence the horses during data collection and previous studies have reported no significant effects of side handling on head and pelvic movement symmetry [38]. As only one force platform was available, an effect of trial may also be present in these data. Recording concurrent forces from multiple force platforms would negate any effect of trial, but to date there are few labs with this capability. Recording GRF when the horses perform other gaits or tasks would also assist in separating out confounding factors from compensatory mechanisms. In hindsight, collecting data at walk would have benefitted this study.

The implications of medio-lateral concomitant GRF production in the hindlimbs of uneven fore footed horses are yet to be established, but are expected to be a risk factor for both hindlimb and sacroiliac / vertebral column orthopedic health. In order to produce a laterally directed GRF the horse must either position the limb more medially during stance, so that the COM provides a lateral directed GRF, and/or medial-lateral hoof wall height may alter the COP origin under the hoof, which would influence the GRF vector direction [27]. Medial hindlimb foot placement could be achieved by adducting the limb further under the body [39], or by the hindquarters rotating in yaw medially prior to stance, due to unbalanced dorsal plane moments. In humans narrower step width is associated with increased medio-lateral force production, increased pronation, greater hip adduction, greater knee internal rotation and increased tibial stress [39,40]. In an equine model of the spine, increasing stiffness along two thirds of the spine increased lateral and dorsoventral peak torques at the next joint to the stiffened spine [41]. Secondary health problems due to unevenness have yet to be fully evidenced, but are expected to be associated with increases in tissue and joint stress, and may include similar anatomical locations to those described here.

To assess the influence of hoof wall medio-lateral height on GRF vector direction in the hindlimbs a post-hoc correlation was performed between hoof wall height difference, based on medio-lateral markers and frontal plane VecAng results (S1 File). No relationship in either hindlimb was found in the UNEVEN group of horses. A more complex analysis of hindlimb hoof asymmetry and functional asymmetry was not possible with these data, as detailed records of hoof shape were not recorded at the time of data collection. Further analysis was performed for the forelimbs using the COP data from [16]. For 11/14 (79%) of the HIGH-RF horses, a lateral COP position was maintained throughout stance in the right forelimb. The frontal plane VecAng was significantly more medial in this limb (Table 4). Only one horse in the HIGH-LF group that was used in their analysis (n = 11) consistently maintained a lateral COP position and in our results, no significant differences were found in frontal plane VecAng in the HIGH-LF group. Although quite convincing in relation to the forelimbs, the effects of COP origin on GRF vector magnitudes should be considered with caution, as in the human literature, pronation, which does influence the position of the COP under the foot, is not strongly linked with medio-lateral GRF production [42]. The influence of foot placement compared to hoof asymmetry on COP location and GRFs in the medio-lateral direction, particularly in the hindlimbs requires further examination.

## Conclusions

This study highlights the three dimensional GRF vector patterns that are produced by uneven footed horses. These include out of sagittal plane GRFs, which were not significantly evident in even footed horses. These results build upon current knowledge of compensatory mechanisms in asymmetric horses, which previously only included kinematics and vertical GRFs. It is evident from increased propulsive and frontal plane GRF production that COM balance is challenged more readily in uneven footed horses and this may impact general orthopaedic health (limbs and vertebral column).

## Supporting information

S1 File Additional data exploring hoof shape, centre of pressure (COP) and vector variables. (XLSX)

S2 File Raw data file. (XLSX)

S3 File SPM data HIGH-LF. (XLSX)

S4 File SPM data HIGH-RF. (XLSX)
